# Enforced Expression of the Transcriptional Coactivator OBF1 Impairs B Cell Differentiation at the Earliest Stage of Development

**DOI:** 10.1371/journal.pone.0004007

**Published:** 2008-12-23

**Authors:** Alain Bordon, Nabil Bosco, Camille Du Roure, Boris Bartholdy, Hubertus Kohler, Gabriele Matthias, Antonius G. Rolink, Patrick Matthias

**Affiliations:** 1 Friedrich Miescher Institute for Biomedical Research, Novartis Research Foundation, Basel, Switzerland; 2 Department of Biomedicine, Division of Developmental Molecular Immunology, University of Basel, Basel, Switzerland; University of Miami, United States of America

## Abstract

OBF1, also known as Bob.1 or OCA-B, is a B lymphocyte-specific transcription factor which coactivates Oct1 and Oct2 on B cell specific promoters. So far, the function of OBF1 has been mainly identified in late stage B cell populations. The central defect of OBF1 deficient mice is a severely reduced immune response to T cell-dependent antigens and a lack of germinal center formation in the spleen. Relatively little is known about a potential function of OBF1 in developing B cells. Here we have generated transgenic mice overexpressing OBF1 in B cells under the control of the immunoglobulin heavy chain promoter and enhancer. Surprisingly, these mice have greatly reduced numbers of follicular B cells in the periphery and have a compromised immune response. Furthermore, B cell differentiation is impaired at an early stage in the bone marrow: a first block is observed during B cell commitment and a second differentiation block is seen at the large preB2 cell stage. The cells that succeed to escape the block and to differentiate into mature B cells have post-translationally downregulated the expression of transgene, indicating that expression of OBF1 beyond the normal level early in B cell development is deleterious. Transcriptome analysis identified genes deregulated in these mice and *Id2* and *Id3*, two known negative regulators of B cell differentiation, were found to be upregulated in the EPLM and preB cells of the transgenic mice. Furthermore, the *Id2* and *Id3* promoters contain octamer-like sites, to which OBF1 can bind. These results provide evidence that tight regulation of OBF1 expression in early B cells is essential to allow efficient B lymphocyte differentiation.

## Introduction

The development of B lymphocytes is under precise control by a large number of transcription factors acting at distinct stages to promote cellular differentiation, survival or proliferation. Critical factors for early B cell specification and commitment are E2A, early B cell factor 1 (EBF1) and Pax5 and other factors play important roles at later stages (reviewed in [1]–[4]). OBF1 is a transcriptional coactivator that is expressed predominantly in B cells but also in activated T cells and forms a ternary complex with the POU domain transcription factors Oct1 and/or Oct2 on conserved octamer motifs (ATGCAAAT) of immunoglobulin (Ig) and other target genes [5]–[9]. The *OBF1* gene encodes a nuclear isoform (p34) and also a cytoplasmic protein (p35) whose function is unclear [10]. While it was initially thought that OBF1 is an essential factor for *Ig* gene transcription [5], analysis of OBF1 deficient mice revealed that in B cells of these mice the level of unswitched Ig μ gene expression is normal [11]–[13], therefore suggesting that this factor must have other target genes. Work from several laboratories has shown that OBF1 has an important function in late B cell development: ablation of OBF1 leads to reduced splenic seeding by transitional B cells and to lower numbers of recirculating B cells in the bone marrow [14], [15]. Furthermore, OBF1 mutant mice have a severely impaired T cell dependent (TD) humoral immune response with low levels of isotype-switched secondary immunoglobulins (IgGs) and *OBF1^−/−^* follicular B cells fail to form germinal centers (GCs) [11], [12], [16], [17]. This absence of GCs may be due in part to the impaired expression of the Ets factor SpiB, which we showed to be a direct target of OBF1 in B cells [18] and is itself important for GC formation [19]. In a pure *C57BL/6* genetic background OBF1 is also crucial for marginal zone (MZ) B cells [20].

Although the first identified functions of OBF1 are found in the periphery, increasing evidence suggests that this factor also plays a significant role at early stages of B cell ontogeny. In the bone marrow OBF1 promotes the survival of transitional B cells [14], [15], and is also critical for V(D)J recombination and transcription of a subset of IgVκ genes [21], thereby having an impact on the IgVκ repertoire [22]. In addition, when the OBF1 mutation is combined with a mutation in the zinc finger transcription factor Aiolos, a severe reduction of the immature B cell pool in the bone marrow is observed that defines a crucial function for OBF1 at the preB2 to immature B cell transition [23], [24]. Intriguingly, a recent study has demonstrated that the cytoplasmic p35 isoform of OBF1 interacts with the tyrosine kinase Syk, thus contributing to regulation of preBCR signaling and preB cell proliferation [25].

Here we have generated transgenic mice expressing the nuclear p34 OBF1 isoform in B cells under the control of the Ig heavy chain variable (*Ig V_H_*) region promoter and μ intron enhancer (*Eμ*). Surprisingly, we observed that these mice have strongly reduced numbers of follicular B cells in the periphery as well as of preB cells in the bone marrow. In addition, these mice show defects in the immune response elicited by follicular B cells, but have a normal MZ B cell response. We present evidence that these defects are due to the premature expression of OBF1 in early progenitors with lymphoid and myeloid potential, the so-called EPLM cells, which normally do not yet express this factor. Furthermore, we identified a number of genes which are deregulated in the transgenic cells, among which the negative regulators *Id2* and *Id3*. Thus, strict control of the level of OBF1 expression during the earliest stage of B cell development is critical for the formation of a functional B cell compartment.

## Materials and Methods

### Mouse strains and transgenic mice generation

The *Eμ-V_H_-OBF1* construct contains a C-terminally HA epitope-tagged human *OBF1* cDNA under the control of the murine *Eμ* enhancer coupled to the *V_H_* promoter from hybridoma 17.2.25. The *Eμ* enhancer was isolated as a 1 kb XbaI fragment from plasmid 1–27 and the *V_H_* promoter was obtained as a 0.2kb fragment from plasmid S-19; additional details of the construction and nucleotide sequence are available upon request. Transgenic mouse lines were obtained and bred in *B6CF1*×*C57BL/6* after which they were intercrossed. All the presented analyses were done with littermates of the different genotypes (WT or BCS). Animal experimentation was carried out according to regulations effective in the Kanton of Basel-Stadt, Switzerland as well as in accordance with the FMI internal regulations under supervision of the FMI Animal Committee. The mice were housed in groups of one to six animals at 25°C with a 12∶12 h light-dark cycle. They were fed a standard laboratory diet containing 0.8% phosphorus and 1.1% calcium (NAFAG 890, Kliba, Basel, Switzerland). Food and water was provided *ad libitum*.

### Splenic B cell purification

The splenic B cells were positively separated with CD19 microbeads following the manufacturer's protocol (Miltenyi Biotec).

### Immunizations

To induce a T-independent antibody response, mice were injected intravenously with 100 µg NIP-Ficoll. Sera were collected from tail bleeding prior to and 10 days after immunization and stored at −20°C.

To induce a T-dependent antibody response, mice were injected subcutaneously with 50 µg alum-precipitated NIP-ovalbumin or DNP-KLH. Sera were obtained from tail bleeding prior to and 14 days after immunization and stored at −20°C.

### ELISA

96-well microplates were coated over night at 4°C with DNP-BSA or NIP-BSA (5 µg/ml in PBS). After extensive washing with PBS the microplates were blocked for 1 hour with ELISA buffer (4% BSA, 0.2% Tween20 in PBS). After extensive washing 3 times serial dilutions of serum samples in ELISA buffer were incubated for 2 hours at room temperature. The serum was removed by extensive washing and alkaline phosphatase-labeled anti-IgM or anti-IgG antibodies (1∶2000, at room temperature for 2 hours) were used as developing reagents. After washing, substrate buffer (100 mg/ml nitrophenylphosphate, 0.1 g/l MgCl_2×_6H_2_O, 10% diethanolamine pH 9.8) was used to reveal bound antibodies. The plates were analyzed on an ELISA reader at 405 nm. All antibodies were from Southern Biotech Associates (Birmingham, AL). The antibody titers were determined by taking the dilutions which correspond to three times the value of the background, considering that it is in the linear phase.

### Real-Time PCR

RNA was purified with the RNeasy Microkit (Qiagen) according to the manufacturer's instructions. cDNA was synthesized with the Thermoscript Reverse Transcriptase Kit (Invitrogen). Quantitative real-time PCR (qPCR) was performed on an ABI PRISM 7000 Sequence Detection System (Applied Biosystems, Foster City, CA) using a SybrGreen-based kit from Eurogene. Normalization was done by amplification of RNA polymerase II (RPII) transcripts.

Primer sequences for qPCR:

OBF1-HA: 5′-CAC TCT CTC TGT GGA AGG CTT TG-3′ and 5′-TTC TCA GCT CTA GAC GGC GTA GT-3′

mOBF1: 5′-CAC GCC CAG TCA CAT TAA AGA A-3′ and 5′-TGT GGA TTT TTG CCA GAG CAT-3′

RPII: 5′-TGC GCA CCA CGT CCA ATG ATA-3′ and 5′-AGG AGC GCC AAA TGC CGA TAA-3′

E2A: 5′-GCA TGA TGT TCC CGC TAC CTG T-3′ and 5′-ACC TTC GCT GTA TGT CCG GCT A-3′

EBF1: 5′-AGA TTG AGA GGA CGG CCT TTG T-3′ and 5′-TCT GTC CGT ATC CCA TTG CTG-3′

PAX5: 5′-AAT CGC TGA GTA CAA ACG CCA A-3′ and 5′-TCC GAA TGA TCC TGT TGA TGG A-3′

Id2: 5′-TCT CCT CCT ACG AGC AGC AT-3′ and 5′-CCA GTT CCT TGA GCT TGG AG-3′

Id3: 5′-ACG ACA TGA ACC ACT GCT ACT CG-3′ and 5′-AGT GAG CTC AGC TGT CTG GAT C-3′

Syndecan1: 5′-GCG GCA CTT CTG TCA TCA AAG-3′ and 5′-GCT GTG TTC TCC CCA GAT GTT T-3′

### Immunofluorescent staining and flow cytometry (FACS) analysis

FACS analysis was performed on a FACSCalibur (BD Biosciences, San Jose, CA). Cell sorting was performed on a MoFlo (DakoCytomation) or on a FACS Aria (BD Biosciences).

FITC-, PE-, APC-, or biotin-conjugated monoclonal antibodies (mAb) specific for B220, CD3, CD4, CD5, CD8, CD11b, CD19, CD21, CD23, CD25, CD43, CD45.2, CD117, IgM, and NK1.1 were purchased from Pharmingen (BD Biosciences), San Diego, CA. Anti-CD117-APC was purchased from e-Bioscience (San Diego, CA). Anti-CD93 (PB493/AA4.1), anti-IgM and anti-IgD antibodies were purified from the hybridoma supernatant and labeled with biotin in our laboratory by standard methods.

For EPLM cell sorting, erythrocyte-depleted bone marrow cells were stained in IMDM 2% FBS with saturating concentrations of anti-B220-FITC, anti-CD19-PE+anti-NK1.1-PE, anti-CD117-APC and biotinylated anti-CD93 antibodies. After 30 min incubation at 4°C, the cells were washed and resuspended in PBS containing streptavidin-PE/Cy7. After a further 30 min at 4°C, the cells were washed, filtered and resuspended at ∼2×10^7^ cells/ml in PBS 2% FBS before sorting.

### Intracellular FACS

After immunostaining of the surface markers, the cells were fixed 10 min with 3% formaldehyde in PBS. The cells were then permeabilized for 10 min with 0.1% Saponin in PBS. After washing with 0.1% Saponin, the cells were incubated 30 min on ice with FITC-coupled anti-HA antibody (Roche). FACS analysis was performed after extensive washing with 0.1% Saponin.

### EPLM cell culture

The OP9 mouse stromal cell line was maintained and expanded in IMDM supplemented with 50 µM β-mercaptoethanol, 1 mM glutamine, 0.03% w/v primatone (Quest, Naarden, The Netherlands), 100 U/ml penicillin, 100 µg/ml streptomycin, and 2% FBS, as described before [26], [27]. OP9 stromal cells were plated 2 days before the addition of sorted EPLMs and were γ-irradiated with 3000 rad at semi-confluency. The culture medium was then replaced by fresh medium supplemented with 100 U/ml IL-7.

### Limiting Dilution Assay

Sorted EPLMs from bone marrow of 3 mice were pooled and plated on semi-confluent γ-irradiated OP9 cells in flat-bottom 96-well plates. Then fresh medium containing ∼100 U/ml IL-7 was added, and 48 replicates with increasing numbers of sorted EPLMs were included. At days 10–14 of culture, all wells were inspected using an inverted microscope. Wells containing colonies of more than 50 cells were scored as positive. The frequency of proliferation was calculated with the L-Calc software. The horizontal line was set at 37% and the vertical lines give the inverse of the frequency as the Poisson law.

### Chimeric mice

5 *C57BL/6* mice were irradiated with 9.5 Gy and 5×10^6^ bone marrow cells (50% from *C57BL/6* mice and 50% from BCS mice) were injected intravenously. After one month, organ cell suspensions were prepared by mechanical disruption, stained, and subsequently analyzed by flow cytometry.

### RNA preparation and hybridization to Affymetrix Microarrays

Cells were FACS sorted and RNA was purified with the RNeasy Microkit from Qiagen. Three individual mice per genotype were used for the EPLM cell sorting. Three WT and four BCS mice were individually used for the Large PreB cell sorting. Each sample was processed independently and ultimately used for one microarray. Total RNA (∼50 ng) from each biological replicate was reverse transcribed and labeled using the Affymetrix 2-cycles labeling kit according to the manufacturer's instructions. Biotinylated cRNA was fragmented by heating with magnesium (as per the Affymetrix instructions) and this fragmented cRNA was hybridized to Mouse 430v2 GeneChips (Affymetrix, Santa Clara, Calif.). Data were analyzed using Expressionist (Genedata AG). The normalized data were subjected to a Student *t*-test (*P*<0.01) and were required to have a median fold change of at least 2. The microarray data has been deposited in Gene Expression Omnibus (GEO) system under the accession number GSE12421.

### Chromatin immunoprecipitation (ChIP)

ChIP was performed with 4.5×10^7^ Abelson B cells as described (Bertolino et al., 2005). Immunoprecipitation was performed with 5 µg of monoclonal OBF-1 antibody C-20 (SC-955 X; Santa Cruz). As a negative control, the chromatin was immunoprecipitated with rabbit IgG (Sigma). The samples were amplified using Taq DNA polymerase using the following primers:

Id2: 5′-TGA CAA AGA GCT TCC CAA GAG-3′ and 5′-CAC GAC AGG TTT AGC GTG AA-3′

Id3: 5′-AGC ACT AGG GAG GCA GAT CA-3′ and 5′-AAA ATC ATG GCC TTC AGT GC-3′

## Results

### Mice overexpressing OBF1 have reduced numbers of follicular B cells

**Figure 1:**
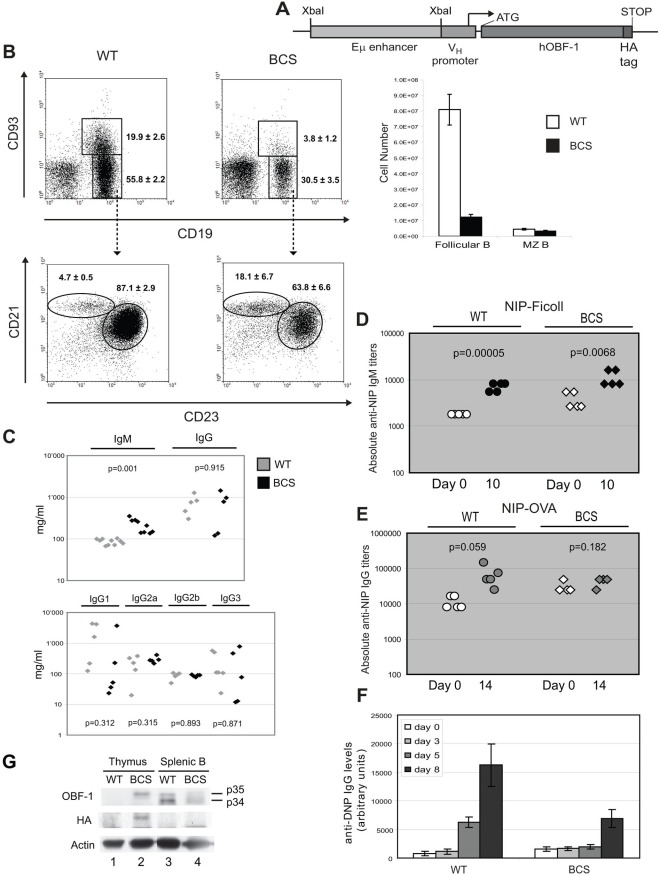
Transgenic *Eμ-V_H_-OBF1* mice are immunodeficient due to decreased numbers of follicular B cells. (A) Schematic of the transgene consisting of an HA-tagged *OBF1* cDNA under the control of the murine *Eμ* enhancer/*V_H_* promoter. The *V_H_* promoter fragment used contains the conserved heptamer and octamer sites as well as the TATA box and the transcription start site. The 1 kb enhancer fragment contains all the known regulatory elements of the *Eμ* enhancer. (B) FACS analysis of splenocytes. Single cell suspensions were stained with antibodies against the indicated markers and representative dot plots are presented. Biotinylated anti-CD93 in combination with streptavidin-PE-Cy7, anti-CD19-APC, anti-CD21-FITC and anti-CD23-PE antibodies were used. The transitional B cells are CD19^+^ CD93^+^, the mature B cells are CD19^+^ CD93^−^; within the mature cells, the MZ B cells are CD21^+^ CD23^low^ and the follicular B cells are CD21^low^ CD23^+^ (left). The absolute number of follicular and MZ B cells is presented (right). Shown values are the mean percentage and absolute number, ±SE, of four individual mice. (C) Immunoglobulin level in unimmunized mice. The level of total IgM, IgG, IgG1, IgG2a, IgG2b, and IgG3 were measured in unimmunized WT and BCS mice. (D) T-independent immune response. The mice were immunized with NIP-Ficoll *i.v.* and serum antigen-specific IgM titers were analyzed by ELISA after 10 days; for each genotype, five mice were analyzed. (E) T-dependent immune response. The mice were immunized with NIP-OVA and serum antigen-specific IgG titers were analyzed after 14 days. Five control and four BCS mice were used. (F) T-dependent immune response. The mice were immunized with DNP-KLH and serum antigen-specific IgG levels were measured at day 0, 3, 5 and 8 after immunization. The histograms represent the mean±SE of three mice per genotype. (G) Endogenous and transgenic OBF1 protein level in thymocytes and splenic B cells. OBF1, HA and Actin were detected by Western blot with thymocytes and splenic B cells of the indicated genotype.

OBF1 expression is largely B cell-restricted, and is modulated during B cell development, with a first peak of expression in the bone marrow at the preB stage and a second peak in germinal center cells of immunized mice [17], [28], [29]. To define whether tightly regulated expression of OBF1 is critical for B cell development and/or function we generated transgenic mice expressing an HA epitope-tagged OBF1 cDNA under the control of an immunoglobulin variable heavy chain promoter and μ heavy chain enhancer (Fig. 1A). This promoter/enhancer combination has been widely used to express transgenes at high level in B cells, with expression starting already very early in B cell ontogenesis [30]–[33]. The transgene was made in such a way that only the p34 nuclear isoform of OBF1[10] is expressed. In reporter assays with transfected cells the human and the mouse OBF1 p34 proteins are equally active and presence of a C-terminal HA tag does not impair function (data not shown). Using this construct three independent transgenic lines were obtained, hereafter called BCS mice, which all exhibited the phenotype described below.

We first examined the peripheral B cell compartment by analyzing splenic B cells with flow cytometry, using combinations of specific antibodies. In the spleen, the newly formed, so-called transitional, B cells are CD93^+^, whereas the mature B cells are CD93^−^ [34]. The mature B cell gate can be further subdivided into the sessile MZ B cells (CD23^low^ CD21^high^) and the follicular B cells (CD23^high^ CD21^low^). Unexpectedly, the BCS transgenic mice showed a strong reduction in the number of splenic transitional and follicular B cells (Fig. 1B, left). The increased relative percentage in the MZ gate by FACS analysis is due to the reduction of the follicular B cell compartment and not to an increase of MZ B cell number (Fig. 1B, right). In line with this, total splenic cellularity is reduced about five fold in BCS mice, with the numbers of B cells and T cells being reduced about 7 fold and 2 fold, respectively (data not shown).

We then measured the level of secreted Igs in the serum of BCS and wild type mice; as shown in Figure 1C, BCS mice have a slightly but significantly, elevated total IgM level, while total IgG levels are not altered. Furthermore, when specific IgG isotypes were examined, no significant difference was observed between BCS and WT mice.

We next monitored the immune response of MZ B cells in BCS mice by injecting them with NIP-Ficoll and measuring the anti-NIP IgM serum titers after 10 days. Indeed, this T-independent immune response was robust in the BCS mice (Fig. 1D), although the basal level of anti-NIP IgM was slightly higher than in the control mice. The immune response of follicular B cells was also investigated by injecting NIP-OVA subcutaneously and measuring the NIP-specific anti-IgG serum titers 14 days later. In this case, this TD immune response was found to be significantly weaker in the BCS than in the control mice (Fig 1E). The impaired T-dependent immune response was further confirmed by immunizing mice with DNP-KLH, another TD antigen, and examining specific IgG serum titers at different time points (Fig. 1F); in this case, a delayed and reduced response in the BCS mice was also observed, in good agreement with the observations presented above.

Next, western blot analysis was performed to investigate the expression of the transgene in splenic B cells and in the thymus, as the promoter/enhancer combination used to drive the *OBF1* cDNA can also be active in T cells [35]. Surprisingly, in splenic B cells of BCS origin expression of the transgenic OBF1 protein was not detected by either the anti-OBF1 or the anti-HA antibody, suggesting that it may be very low (see also below). In contrast, in WT splenic B cells bands corresponding to the p34 and p35 isoforms of endogenous OBF1 were detected (Fig. 1G, lanes 3 and 4). Moreover, in thymocytes from BCS, but not from WT mice, transgenic OBF1 expression could be evidenced by both antibodies. Since the OBF1 expressed from the transgene should be an HA-tagged p34 protein, its expected size is marginally larger than that of endogenous p35 and this is just what is observed (compare lanes 2 and 3). Together, the reduced follicular B cell numbers, the apparent lack of OBF1 expression in mature B cells and the T dependent immunodeficiency observed in BCS mice suggest the presence of a defect at an early developmental stage in the bone marrow.

### The decrease of splenic B cell populations is due to impaired early B cell differentiation

**Figure 2:**
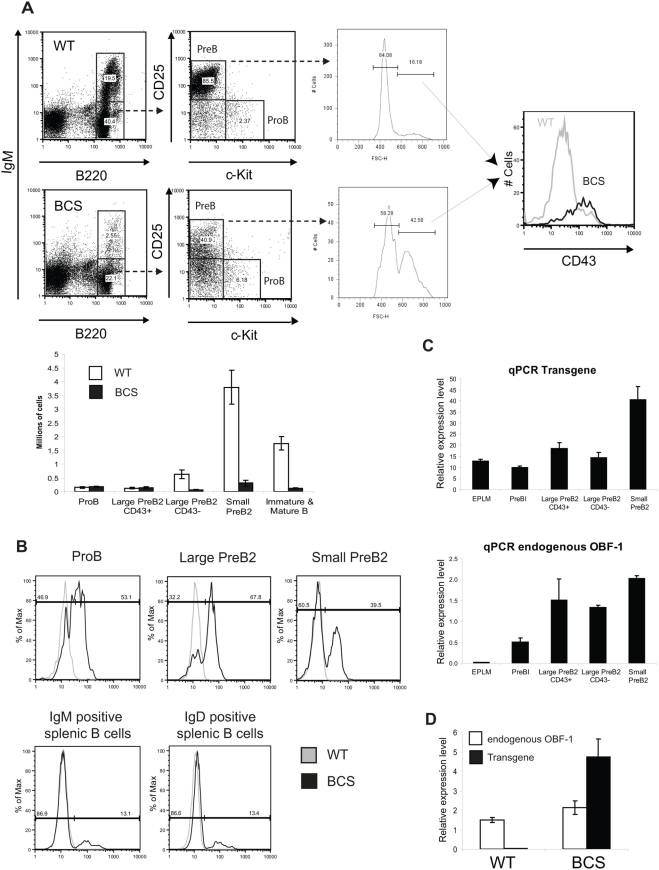
Enforced expression of OBF1 impairs B cell differentiation at the earliest stage. (A) FACS analysis of bone marrow cells. B cells were labeled with an anti-B220-APC antibody and the stages of differentiation were identified with anti-c-kit-PE, biotinylated anti-CD25 in combination with streptavidin-PE-Cy5.5, anti-CD43-PE, and anti-IgM-FITC. The CD43 expression profile is shown specifically for the large preB2 cells (upper right). Representative dot plots are shown and the histogram with the cell numbers presents the mean±SE in the different fractions based on three individual mice of each genotype. (B) Intracellular expression of the transgenic OBF1 protein was detected with an anti-HA-FITC antibody in combination with various B cell stage-specific antibodies: In the bone marrow proB cells were detected with anti-B220-APC and anti-c-kit-PE, preB2 cells were labeled with anti-B220-APC together with biotinylated anti-CD25 combined with streptavidin-PE-Cy5.5 and further discriminated for size; splenocytes were labeled with anti-B220-APC, together with either biotinylated anti-IgM or anti-IgD in combination with streptavidin-PE-Cy5.5. (C) qPCR of endogenous and transgenic OBF1 RNA from the indicated bone marrow cell populations; the EPLM and preB1 populations were identified and isolated as described in Figure 3. The histograms represent the mean±SE of three individual mice for the EPLM and preB1 cells and two individual mice for the large and small preB2 cells. (D) qPCR of endogenous and transgenic OBF1 RNA from splenic B cells. The histogram represents the mean±SE of two individual mice.

To identify the cause of the reduced splenic B cell compartment in BCS mice, the bone marrow B cell populations were investigated by FACS analysis using a number of antibodies allowing to define the early stages of B cell development. The cellularity of the total bone marrow is reduced by about 10 % in the BCS mice. Among the B220^+^ cells, the IgM negative and positive gates contain the preB/proB cells and the immature/mature B cells, respectively. Furthermore, within the IgM negative cells, expression of c-kit and CD25 can be used to distinguish the proB and the preB cells; in WT mice, the vast majority of CD25^+^ preB cells are small and quiescent and derive from large cycling cells [36]. As shown in Figure 2A, BCS mice show a strong decrease in the number of CD25^+^ preB cells, and a relative increase in the proportion of the large preB cells. Furthermore, this latter population has predominantly a high CD43 staining, indicating that the impaired differentiation occurs within the large preB cell stage, at the transition between CD43^+^ and CD43^−^ (Fig. 2A, right panel). It results that all the downstream populations, immature and mature recirculating B cells (B220^+^ IgM^+^), are strongly reduced in these mice.

Intracellular FACS analysis with an α-HA antibody was performed to investigate the expression of the transgene during B cell development. As shown in Figure 2B, transgenic OBF1 protein is well expressed until the large preB cell stage and is gradually downregulated in cells that have passed this developmental stage, resulting in a dramatic loss of expression in mature splenic B cells in good agreement with the western blot data presented in Figure 1G. Unlike the protein, the transgene RNA is expressed from the earliest stage examined (EPLM, see below) and its expression remains relatively constant throughout B cell differentiation (Fig. 2C), including in splenic B cells (Fig. 2D), indicating that the downregulation of OBF1 protein takes place at the post-transcriptional level. In contrast, in WT mice endogenous OBF1 RNA is not detectable in EPLMs and shows a low level of expression in preB1 cells, followed by higher expression starting at the large preB2 cell stage (Fig. 2C).

### The EPLMs have a strong B cell commitment deficiency in BCS mice

At a first glance the proB cell population (B220^+^ c-kit^+^) is normal in the BCS mice. However proB cells form an heterogeneous population, which in majority contains already committed B cells (preB1: CD93^+^ CD19^+^), but also uncommitted progenitors of several kinds, including NK1.1 positive cells and others. Within these uncommitted progenitors a significant fraction of the cells are “early precursors with lymphoid and myeloid potential”, so-called EPLMs (CD93^+^ CD19^−^ NK1.1^−^). These cells, while not committed yet to the B cell lineage, under normal conditions preferentially become B cells *in vivo*, are able to generate T cells under transplantation conditions and also have the capacity to differentiate *in vitro* along the myeloid pathway [26].

**Figure 3:**
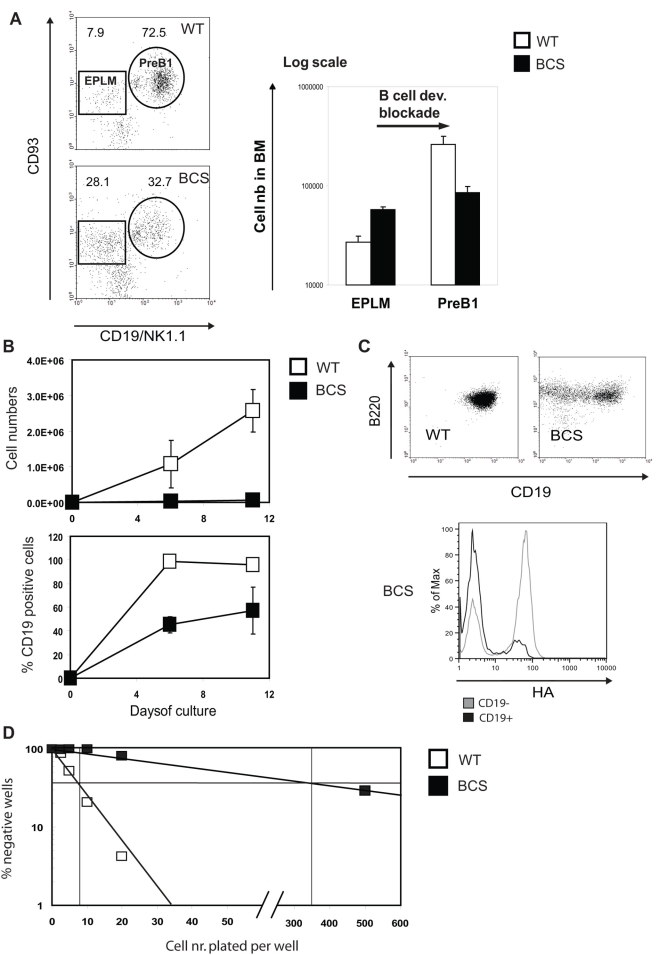
Differential block after the EPLM cell stage and impaired B cell commitment. (A) Detailed FACS analysis of the proB cell compartment in the bone marrow. ProB cells were detected by labeling bone marrow cells with anti-B220-FITC and anti-c-kit-APC. The proB cell gate was then subdivided into EPLM and preB1 cells on the basis of staining with anti-CD19-PE/anti-NK1.1-PE and biotinylated anti-CD93 in combination with streptavidin-PE-Cy7 (left). The cell number of the different bone marrow populations is presented in the histogram with the mean±SE of three individual mice (right). (B) *In vitro* culture of EPLMs on OP9 feeders. 10'000 EPLMs from pooled mice (n = 3) were plated on OP9 feeders in a 24 well microplate. Cell number and percentage of cells positive for CD19 expression were determined at the indicated times. The graphs represent the mean±SD from 2 independent samples per time point. (C) Expression of CD19 in cultured EPLMs was determined at day 11 by staining with anti-B220-APC and anti-CD19-PE; expression of the transgene was examined by additional intracellular staining with anti-HA-FITC (lower part). (D) LDA for B cell commitment of EPLMs cultured on OP9 feeders. WT or BCS EPLMs were cultured at increasing dilutions and the number of positive wells was determined under an inverted microscope after 11 to 14 days.

We therefore examined these populations and surprisingly observed that the EPLMs are strongly increased in percentage and number while the preB1 cell numbers are reduced in the BCS mice, indicating an initial differentiation block at this stage already (Fig. 3A). We then sorted EPLMs from WT and BCS mice and tested their capacity to expand and differentiate *in vitro* under culture conditions promoting B cell growth. As shown in Figure 3B, the BCS EPLMs expand very slowly and their differentiation, monitored by the appearance of CD19 expression, is significantly impaired. Intracellular FACS analysis of the EPLM cultures indicated that the cells that succeed to upregulate CD19 also downregulate the OBF1 transgene (Fig. 3C), much like what had been observed in early B cells progressing through developmental stages *in vivo*. Next, limiting dilution assays (LDA) of EPLMs on OP9 feeders were performed to compare, in EPLM cell populations of WT or BCS origin, the frequency of precursors capable of establishing a colony [27]. In this assay, the WT cells showed a normal frequency (1/8), while the BCS cells had a dramatically lower frequency (1/346; Fig. 3C). Together these results demonstrate that the BCS cells are impaired in their B cell commitment potential.

### The differentiation block is intrinsic to B cells

**Figure 4:**
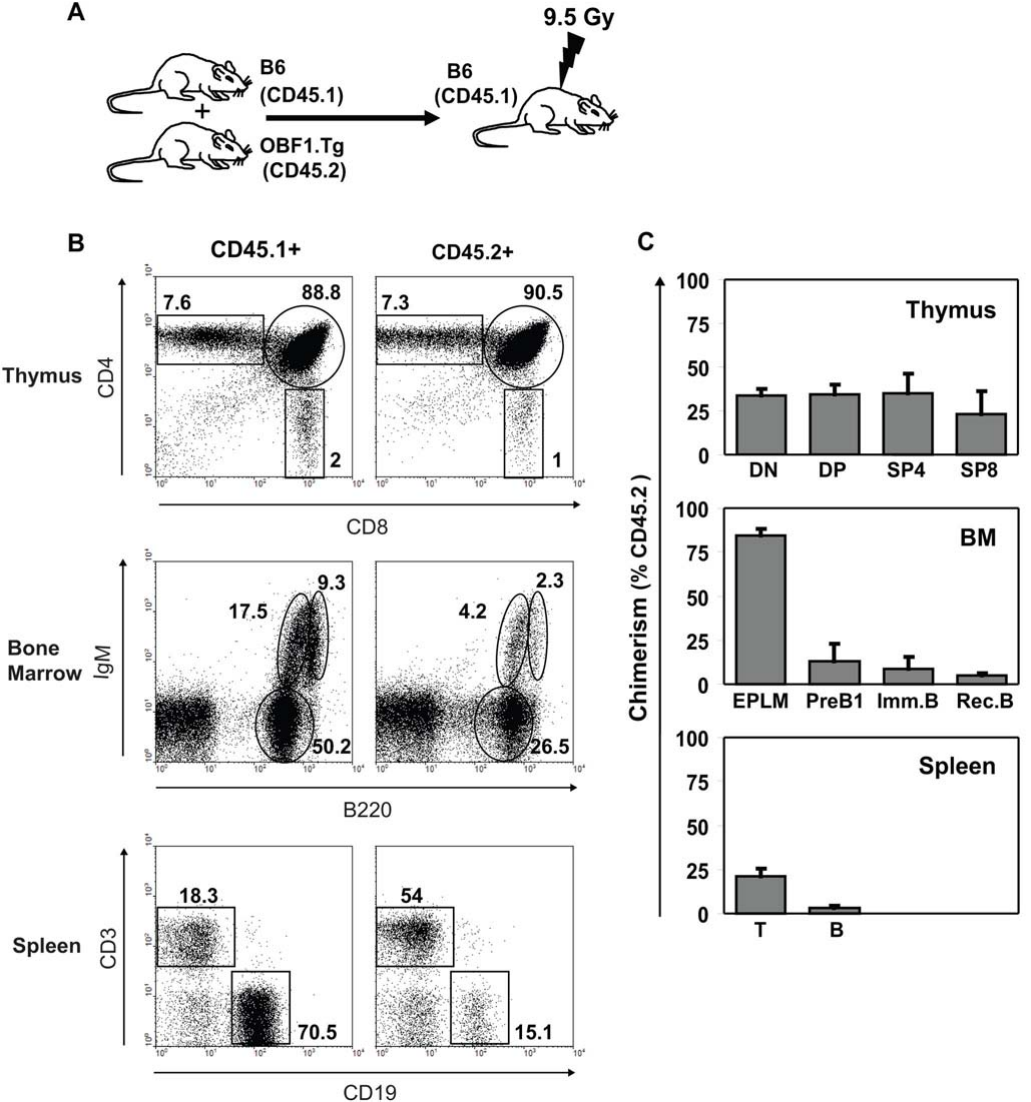
The differentiation block is intrinsic to B cells. (A) The experimental strategy for the mixed bone marrow chimera is depicted. (B) FACS analysis of thymus, bone marrow and spleen of reconstituted mice. Thymocytes were stained with anti-CD4-FITC and anti-CD8-PE. Bone marrow B cells were stained with anti-B220-PE and anti-IgM-APC. Splenocytes were stained with anti-CD3-PE and anti-CD19-APC. In addition, cells of BCS origin were stained with an anti-CD45.2 (Ly5.2) conjugated to either APC or FITC. (C) Percentage of chimerism in the thymus, the bone marrow and the spleen. The EPLM and preB1 cells were analyzed using anti-B220-FITC, anti-CD19-PE, anti-NK1.1-PE, anti-cKit-APC and biotinylated anti-CD45.2 combined with streptavidin-PE-Cy7 antibodies. BM, bone marrow. DN, double negative cells; DP, double positive; SP4, single positive CD4^+^; SP8, single positive CD8^+^. Imm. B, immature B cells; Rec. B, recirculating B cells.

The experiments presented so far demonstrate that enforced OBF1 expression in EPLMs impairs their differentiation potential and leads to a developmental block: only cells that successfully downregulate the transgene can differentiate normally along the B cell pathway. To investigate whether the differentiation defect is intrinsic to B cells, competitive chimera mice were generated. For this, the bone marrow of BCS mice (CD45.2, aka Ly5.2) and “competitor” bone marrow from *C57BL/6* mice (CD45.1, aka Ly5.1) were mixed at a 50∶50 ratio and used to inject into γ-irradiated mice having the same haplotype as the competitor (CD45.1; Fig. 4A). The reconstituted mice were then sacrified one month post-injection and analyzed. The developing T cell compartment of the chimera mice was not affected, as seen by examining the expression of CD4 and CD8 on thymocytes: for all the developmental stages examined ca. 30% of the thymocytes were BCS-derived. Likewise, in the spleen about 25% of the T cells were of BCS origin. In contrast, the B cell compartment of the BCS haplotype (CD45.2) was strongly impaired in the bone marrow and also in the spleen, as had been initially observed in the BCS mice (Fig. 4B). Indeed, the bone marrow of reconstituted mice showed a clear block at the EPLM-preB1 transition, and the chimerism percentage was found to be inverted just between these two stages: 80% of the EPLMs, but only 10% of the preB1 cells, were BCS-derived (Fig. 4C). These results demonstrate that the differentiation deficiency is intrinsic to the B cells and not due to the environment in the stroma.

### The negative regulators *Id2* and *Id3* are OBF1 direct target genes

**Figure 5:**
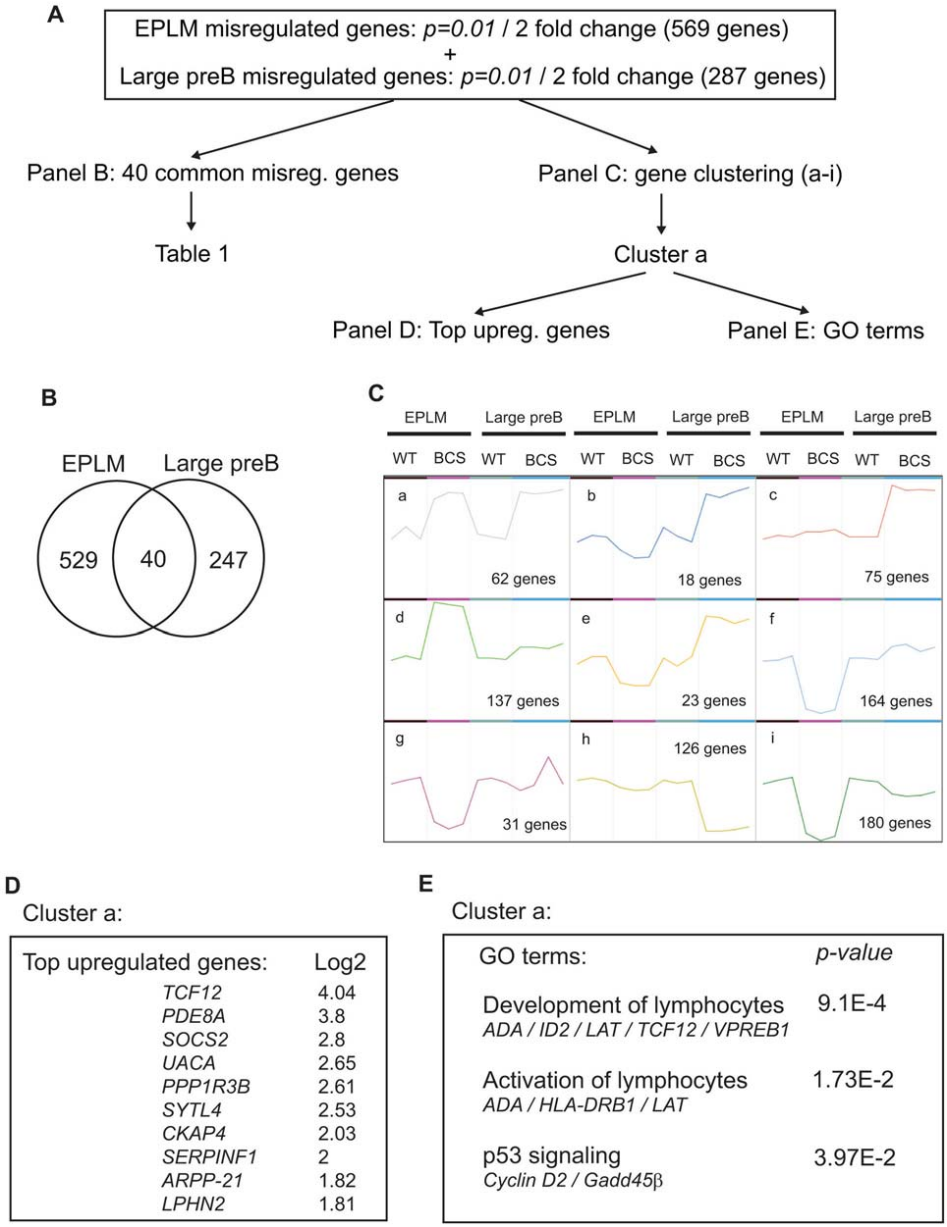
Microarray analysis of EPLM and large preB2 cells. (A) Scheme for analysis of the microarray data. (B) Venn diagram representing the genes that are misregulated at least 2 fold with a P value of 0.01 in EPLM and large preB2 cells. (C) Gene clustering. The deregulated genes were clustered in 9 families according to their expression patterns. (D) Top upregulated genes from the cluster “a” with the upregulation level monitored in EPLM cells. (E) Gene Ontology (GO) terms in the cluster “a”.

To get an insight in the molecular origin of the differentiation blocks caused by OBF1 overexpression, the transcriptome of EPLM and large preB2 cells of each genotype was determined by microarray analysis. The scheme for analysis of the microarray data is depicted in Fig. 5A. We considered genes misregulated at least 2 fold with a stringent P-value of 1%; with these criteria, 569 genes were deregulated in EPLMs and 287 in large preB2 cells, with 40 genes overlapping between the two populations (Fig. 5B). The genes common to EPLMs and large preB2 cells are presented in Table 1.

**Table 1:** List of the genes that are deregulated both in EPLMs and preB1 cells of BCS origin; the cluster corresponding to their expression pattern (Fig. 5C) is indicated.

Accession number	Name, description	Cluster
1427670_a_at	Transcription factor 12	a
1445093_at	Transcription factor 12	a
1417168_a_at	ubiquitin specific peptidase 2	a
1417336_a_at	synaptotagmin-like 4	a
1417460_at	interferon induced transmembrane protein 2	a
1417976_at	adenosine deaminase	a
1418294_at	erythrocyte protein band 4.1-like 4b	a
1418406_at	phosphodiesterase 8A	a
1418507_s_at	suppressor of cytokine signaling 2	a
1419028_at	cyclic AMP-regulated phosphoprotein, 21	a
1425553_s_at	huntingtin interacting protein 1 related	a
1426755_at	cytoskeleton-associated protein 4	a
1448390_a_at	dehydrogenase/reductase (SDR family) member 3	a
1449109_at	suppressor of cytokine signaling 2	a
1460651_at	linker for activation of T cells	a
1432886_at	RIKEN cDNA 5730488B01 gene	a
1459847_x_at	glial cell line derived neurotrophic factor family receptor alpha 2	a
1452985_at	uveal autoantigen with coiled-coil domains and ankyrin repeats	a
1456772_at	neutrophil cytosolic factor 1	b
1434248_at	protein kinase C, eta	c
1439494_at	solute carrier family 5 (sodium/glucose cotransporter), member 9	d
1445169_at	gb:BM232503 /DB_XREF = gi:17867773 /DB_XREF = K0324B04-3	d
1446294_at	Transcribed locus	d
1421908_a_at	transcription factor 12	d
1439619_at	transcription factor 12	d
1449455_at	hemopoietic cell kinase	d
1429001_at	pirin	d
1458802_at	human immunodeficiency virus type I enhancer binding protein 3	e
1434572_at	histone deacetylase 9	f
1423104_at	insulin receptor substrate 1	h
1416762_at	S100 calcium binding protein A10 (calpactin)	h
1456642_x_at	S100 calcium binding protein A10 (calpactin)	h
1418102_at	hairy and enhancer of split 1 (Drosophila)	h
1419481_at	selectin, lymphocyte	i
1433741_at	CD38 antigen	i
1452679_at	tubulin, beta 2b	i
1447807_s_at	pleckstrin homology domain containing, family H (with MyTH4 domain) member 1	i
1455646_at	RIKEN cDNA 2010004M13 gene	i
1415943_at	syndecan 1	i
1437279_x_at	syndecan 1	i

All deregulated genes were then clustered in 9 expression pattern families with the Expressionist program (Fig. 5C). The cluster “a” is possibly the most interesting group, as these genes are upregulated both in EPLM and large preB2 cells of BCS mice, and are therefore putative OBF1 direct target genes. The top upregulated genes in this cluster are presented in Figure 5D; based on the gene ontology (GO) classification, the genes in cluster “a” are mainly involved in lymphocyte development and activation (Fig. 5E). Furthermore, p53 signaling is also affected, as evidenced by the deregulation of the *Cyclin D2* and *Gadd45β* genes (Fig. 5E). In addition, cluster “a” also contains the *Id2* gene, which encodes an inhibitor of the basic helix-loop-helix (bHLH) protein E2A. Cluster “d”, which corresponds to genes specifically deregulated in EPLMs, is also particularly interesting, as it contains another Id gene, *Id3*. Thus, *Id2* and *Id3* are both upregulated in EPLMs of BCS mice and *Id2* is also upregulated in BCS large preB2 cells.

The same gene can appear in different clusters (e.g. *TCF12* in clusters a and d), because different probes recognize different forms of the corresponding gene transcript. However, the meaning of these different transcripts is often not well understood.

We next examined the microarray data for the main transcription factors critical for early development, such as *E2A*, *Pax-5* or *EBF1*; while *E2A* expression was similar in BCS and wild type cells, expression of *Pax-5* and *EBF1* was significantly elevated in BCS EPLMs (cluster “d”). Finally, we found in cluster “i” the *Syndecan1* gene, which is downregulated both in EPLM and large preB2 cells (Fig. 5C). This gene, whose upregulation is often used as a marker for plasma cell differentiation, has been reported earlier to show higher expression at the surface of *OBF1^−/−^* B cells [37].

**Figure 6:**
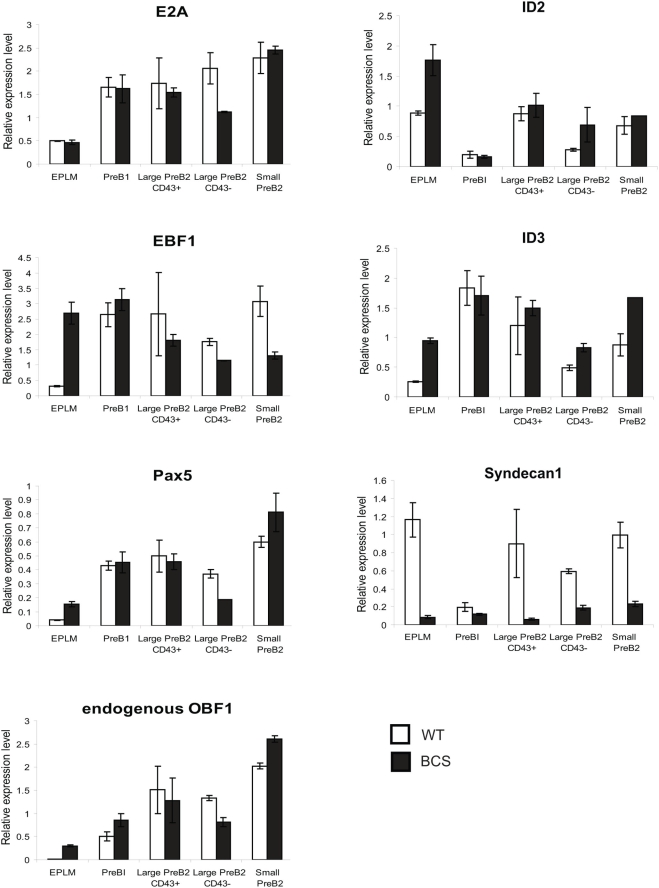
qPCR analysis of early B cell populations. Quantitative RT-PCR analysis of *E2A*, *EBF1*, *Pax5*, endogenous *OBF1*, *Id2*, *Id3* and *Syndecan1* expression in the indicated cell populations. The histograms represent the mean±SE of three individual mice for the EPLM and preB1 cells and two individual mice for the large and small preB2 cells.

To validate these observations we set up quantitative reverse transcriptase PCR reactions with RNA isolated from cells of different developmental stages: EPLMs, preB1 cells, CD43 positive or negative large preB2 cells and also small preB2 cells. As shown in Figure 6, most of the microarray results could be verified in these experiments. *Id2* and *Id3* were found overexpressed in EPLMs expressing OBF1, and also to a lesser extent in large preB2 CD43^−^ cells, the two stages where developmental blocks had been identified. *Pax-5* is upregulated in transgenic EPLMs and also slightly downregulated in large preB2 CD43^−^ cells. Furthermore, BCS EPLMs show a ca. 5 fold upregulation of *EBF1* and also a robust upregulation of endogenous *OBF1* expression; the latter could be caused by the elevated EBF1 expression, as it has recently been shown that this factor directly regulates OBF1 expression in progenitors [38]. Furthermore, *Syndecan1* is downregulated in all the early B cell populations of the BCS mice (Fig. 6), further confirming that it is negatively regulated by OBF1.

**Figure 7:**
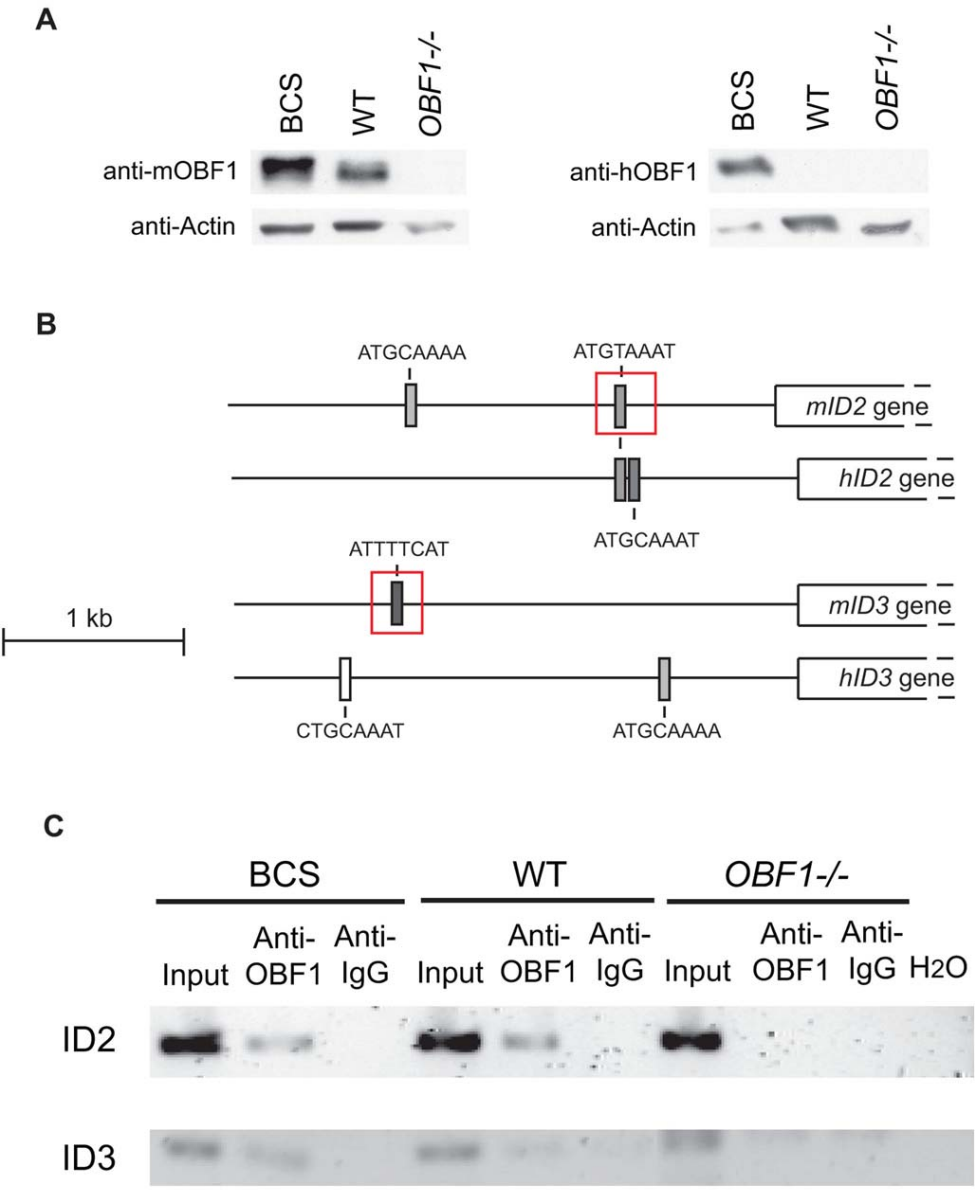
OBF1 can bind to the *Id2* and *Id3* promoter. (A) OBF1 protein level in Abelson (Abl) preB cell lines of different genotypes. PreB cell cultures were established from fetal livers by transformation with the Abelson murine leukaemia virus. Murine OBF1, human OBF1 and Actin were detected by Western blot with protein extracts from the indicated cells. (B) Octamer-like sites in the mouse *Id2* and *Id3* promoter. The relative locations of the motifs with respect to the translation start codon are as follows: mouse *Id2*: −835, −2068; human *Id2*: −1048, −1067; mouse *Id3*: −2200; human *Id3*: −760, −2518. (C) Chromatin immunoprecipitation assay with the *Id2* and *Id3* promoters. ChIP using anti-OBF1 antibody was performed from the BCS, WT and *OBF1^−/−^* Abl cell lines and promoter DNA was amplified as depicted by the red boxes in Fig. 7C.

Since the *Id2* and *Id3* genes are in gene clusters corresponding to putative OBF1 direct targets (Fig. 5C) we searched for potential binding sites in their regulatory region, using the Transcription Element Search System (TESS, http://www.cbil.upenn.edu/cgi-bin/tess/tess). As presented in Figure 7B, the human and the mouse *Id2* and *3* genes contain several elements with homology to the conserved octamer motif found in *Ig* promoters. Furthermore, one of these elements is conserved in sequence and location between the human and mouse *Id2* promoter. Abelson cell lines derived from BCS, WT and *OBF1^−/−^* mice were used to investigate the interaction between OBF1 and the respective octamer sites in the *Id2* and *Id3* promoters. As expected, the Abelson cell line from BCS mice express strongly the transgenic protein (Fig. 7A). Chromatin immunoprecipitation (ChIP) was performed with an anti-OBF1 antibody and DNA fragments encompassing the putative binding sites (depicted by red boxes in Fig. 7B) were amplified by PCR. As shown in Figure 7C, OBF1 was found to interact with the *Id2* and *Id3* promoters in BCS and also in WT cells, but not in OBF1 deficient cells. Furthermore, additional analysis also identified octamer-like motifs in the *EBF1* promoter and preliminary ChIP assays demonstrated OBF-1 binding in BCS but not in WT Abelson cells (data not shown).

## Discussion

Here we present evidence that overexpressing OBF1 at a very early stage of B cell ontogeny is deleterious for B cell development. This misregulated expression pattern of OBF1 ultimately has a dramatic impact on mature B cells in the spleen, as the mice have an impaired T-dependent immune response accompanied with a strong reduction of follicular B cells (Fig. 1Band E, F). This immunodeficiency is likely due to the reduced number of follicular B cells, since the T-independent immune response is not impaired (Fig. 1C). Surprisingly, the number of marginal zone B cells is not affected despite the B cell developmental block in the bone marrow and the decrease in splenic immature B cells. One explanation might be that the transitional B cells entering the spleen first repopulate the MZ compartment and then the follicles, and are in sufficient number to fill the MZ. This hypothesis is supported by the earlier observation that the MZ compartment is normal in several other lymphopenic mutant mice such as *IL-7^−/−^* mice [39] or *Lambda5^−/−^* mice [40]. Furthermore, the response to immunization with NIP-Ficoll, which is known to be dependent on the MZ B compartment [41], is also normal in BCS mice. Thus, by these two criteria the follicular and MZ B cell compartment are differentially affected by the presence of the transgene. The total IgM level in unimmunized BCS mice was higher than in the WT mice suggesting that it could be the cause for the high NIP specific IgM background (Fig. 1C). In fact, the number of B1b B cells is increased in the peritoneal cavity of BCS mice (Figure S1), which could contribute to explain the higher IgM level in these mice. On the other hand the total IgG level, as well as specific IgG isotypes, were not altered in unimmunized BCS mice (Fig. 1C). The transgene is expressed in the thymus at the protein level, but surprisingly not in splenic B cells; this suggests that the cause for the decreased number of immature and follicular B cells is localized at an earlier B cell developmental stage in the bone marrow (Fig. 1G). OBF1 was recently reported to also function in determining T helper cell polarity[42]. Therefore, the elevated OBF1 expression in thymocytes might influence TD antibody responses; however, it is worth noting that T cell development and the numbers of CD4 and CD8 T cells in the thymus are not affected by the transgene (data not shown).

Investigation of the bone marrow, which is the site of early B cell development, allowed to identify the cause of the reduced splenic B cell numbers. A first block was detected between the EPLM and the preB1 cell stage. In the normal situation, EPLMs are mostly committed to the B cell lineage [26] and do not yet express OBF1 (Fig. 2C). However, enforcing expression of OBF1 in this population induces an accumulation of EPLMs with a strong B cell commitment deficiency (Fig. 3). Indeed, the cells that succeed to pass this developmental block downregulate the transgene post-translationally, indicating that the level of OBF1 has to be low at this stage for proper B cell differentiation. OBF1 is known to be regulated at the protein level in mature B cells, potentially through interaction with the Ring finger protein SIAH [43], [44]. Our results suggest that the OBF1 protein level may also be modulated in early B cells, at least in the case of the transgenic mice described here. Whether this modulation of OBF1 protein levels is mediated by SIAH, or by other mechanisms, is not known. Remarkably, *in vitro* cultures of EPLMs showed that premature expression of OBF1 in this cell compartment severely impairs their proliferation and differentiation potential (Fig. 3). This is in stark contrast to the effect observed in OBF1 deficient IL-7 dependent pro-preB cells: in this case, cellular proliferation is markedly improved in comparison to WT cells [25] and data not shown). Thus, in very early B cells OBF1 appears to antagonize cell proliferation and fine regulation of its expression level may be used to set a regulatory threshold. A second differentiation block was also observed after the large preB2 (CD43^+^) cell stage. It is not clear whether this is directly due to the increased expression of OBF1 in the preB cells, or whether this is a secondary effect whose origin is in the EPLMs. Generation of mice overexpressing OBF1 starting at the preB1 or preB2 stage might allow to address this point.

Mixed bone marrow chimera mice could fully recapitulate the initial phenotype and confirmed that the differentiation defect is intrinsic to the BCS B cell precursors (Fig. 4). When a 1∶1 mix of WT and BCS bone marrow was injected into irradiated mice, we observed that about 40% of the thymic developing T cells were of BCS origin and in the spleen the proportion was still about 25%. In striking contrast, the BCS-derived B cell compartment was underrepresented and contained only a few percent of mature B cells in the spleen. Furthermore, a strong developmental block was evident in the bone marrow with a dramatic accumulation of EPLMs accompanied with a deficit to progress to the preB1 stage.

How the deregulated expression of OBF1 in the early EPLM compartment leads to the defects described here is not understood yet. As a first attempt to address this question, we have analyzed the transcriptome of EPLM and large preB2 cells in WT or BCS mice (Fig. 5). We found significantly more genes misregulated at the EPLM stage than at the large preB2 cell stage and relatively little overlap between the two sets of genes. However, several of the main transcription factors and known regulatory molecules of early B cell differentiation were either not affected or rather expressed at a slightly higher level in the BCS-derived EPLMs. For example, the helix-loop-helix factor *E2A* is expressed at a normal level, while *EBF1* and *Pax5* are both upregulated (Fig. 6). Generally our results point to the critical importance of maintaining proper regulation of OBF1 expression during early B cell differentiation. So far, relatively little is known about how the *OBF1* gene is regulated and the DNA sequences controlling its cell-specific and temporal expression have not been delineated yet. A functionally important cAMP response element (CRE) binding site has been identified in the proximal OBF1 promoter [45] but it can not explain the regulated B cell-specific expression of this gene and in transfection experiments the *OBF1* promoter does not appear to be clearly B cell-specific [46]. Interestingly, EBF1 has been very recently identified as a potentially direct regulator of OBF1 expression in progenitors [38]. As shown here, OBF1 is co-expressed from the preB1 stage onwards together with transcription factors like EBF1 that drive B cell commitment (Fig. 2C and Fig. 6). However, in the BCS mice the OBF1 transgene is expressed already before EBF1 and this altered sequence of expression compromises the development of the proB cells. In fact, the elevated level of endogenous OBF1 expression in EPLMs of BCS mice may be a direct consequence of the EBF1 upregulation (Fig. 6), in agreement with the findings of Zandi et al. (2008). However, although EBF1 and Pax5 are misregulated in BCS-derived EPLMs, this does not explain the observed B cell commitment defect, as enforced expression of these genes favours B cell differentiation [47], [48]. Interestingly, *EBF1* showed the same pattern of expression as *Id2* and *Id3* in EPLM cells from BCS mice. The *EBF1* promoter also contains conserved octamer sequences, and preliminary results suggest that they can be targeted by OBF1. Therefore the EBF1-OBF1 axis constitutes a positive feedback loop, as OBF1 is itself an EBF1 target gene. The upregulation of EBF1 in BCS mice may also be explained by the upregulation of Pax5 in EPLM cells, which was recently reported to activate the proximal promoter of *EBF1* (Roessler et al., 2007).

The *Id2* and *Id3* genes were both found deregulated in the microarray as well as in the qPCR validation experiments (Figs. 5 and 6); in particular, elevated expression was found in EPLMs and also in large preB2 cells, which are just the stages were the developmental blocks have been observed in BCS mice *in vivo*. Expression of *Id3* has been reported to be repressed by OBF1 in a preB cell line expressing an inducible OBF1-ER fusion protein [42]; the reason for this difference is not clear, but might be due to differences between the expressed proteins (OBF1 vs OBF1-ER fusion) or between the cells examined. The identification of several motifs with homology to the octamer site in the *Id2* and *Id3* promoters suggested that these genes could be direct OBF1 targets and chromatin immunoprecipitations showed that OBF1 can indeed bind to these octamer sites (Fig. 7). Generally Id proteins have been found to antagonize the activity of bHLH proteins, and in particular of E2A. Several previous studies have shown that low levels of Id proteins are necessary to allow E2A to drive B cell commitment [49], [50]. In line with this, constitutive expression of Id proteins downstream of the preB1 stage was found to impair B cell development, indicating that Id downregulation is critical for B cell ontogeny [51]. Furthermore, it was also reported that Id3 inhibits the growth and survival of B lymphocyte progenitors [52]. These observations therefore suggest that elevated expression of Id2 and Id3 could result in a differentiation block at the EPLM and large preB2 cell stage, as seen in the BCS mice.

Finally, Syndecan1 is a plasma cell marker whose *in vivo* function is not clear yet. However, Syndecan1 was reported previously to be upregulated on the surface of *OBF1^−/−^* splenic B cells [37] and our microarray analysis of *OBF1^−/−^* mice showed that Syndecan1 is upregulated also at the mRNA level (data not shown). Interestingly, we found here that Syndecan1 expression was strongly downregulated in all the early B cell populations of the BCS mice (Fig. 6), indicating that there is a negative correlation between OBF1 and Syndecan1 expression. Thus, *Syndecan1* represents a novel OBF1 target gene.

## Supporting Information

Figure S1 (A) FACS analysis of the spleen and peritoneal cavity. B cells were labeled with an anti-CD19-PE antibody. The B2 cells were stained with anti-CD23-FITC. The B1a cells were stained with anti-CD5-FITC antibody or with anti-CD11b-FITC antibody. (B) B1, B1a, B1b and B2 B cell populations in the spleen and peritoneal cavity. The B2 and MZB/B1 B cells are CD19+CD23+ and CD19+CD23- populations respectively. The B1a and B1b B cells are CD19+CD5+ and CD19+CD23-CD5- populations respectively. The histograms represent the mean±SD of three individual mice per genotype.
(2.58 MB EPS)
